# LncRNA *CCDC152* coordinates a tumor-associated selenoprotein P cycle that sustains selenium-dependent redox homeostasis in glioblastoma

**DOI:** 10.1016/j.redox.2026.104438

**Published:** 2026-10-12

**Authors:** Stephanie Siu, Hiroki Taguchi, Xi Zheng, Hikari Sugiura, Shota Yamashita, Tatsuya Takahashi, Masato Kosuge, Takayuki Kaneko, Misaki Shimizu, Yoshiteru Shimoda, Kota Noritsugu, Kotoko Arisawa, Mika Watanabe, Takashi Toyama, Masayuki Kanamori, Hidenori Endo, Yoshiro Saito

**Affiliations:** a Laboratory of Molecular Biology and Metabolism, Graduate School of Pharmaceutical Sciences, Tohoku University, 6-3 Aoba, Aramaki, Aoba-ku, Sendai, Miyagi, 980-8578, Japan; b Department of Neurosurgery, Graduate School of Medicine, Tohoku University, 1-1 Seiryo Aoba-ku, Sendai, 980-8574, Japan; c Department of Pathology, Tohoku Kosai Hospital, 2-3-11 Kokubuncho, Aoba-ku, Sendai, Miyagi, 980-0803, Japan

**Keywords:** Selenium, Selenoprotein P, LRP8, CCDC152, Ferroptosis, Glioblastoma

## Abstract

Selenium is essential for the synthesis of redox-active selenoproteins, including glutathione peroxidase 4 (GPX4), a central suppressor of ferroptosis. However, how malignant cells coordinate selenium acquisition and reutilization to maintain redox homeostasis remains poorly understood. Here, we identify a tumor-associated selenoprotein P (SELENOP) cycle in glioblastoma (GBM) that is coordinated by the long non-coding RNA (lncRNA) *CCDC152*. Highly proliferative GBM cells secrete SELENOP, recapture it through LRP8, and reutilize selenium to maintain intracellular selenoprotein abundance and cell proliferation. Disruption of SELENOP or LRP8 suppressed GBM cell proliferation, while LRP8 depletion reduced GPX1 and GPX4 abundance and its intracellular domain was required for growth-promoting activity. Unexpectedly, although *CCDC152* suppresses SELENOP translation in hepatocyte-derived cells, it positively regulated SELENOP production, LRP8 expression, and intracellular GPX1 and GPX4 abundance in GBM cells. *CCDC152* depletion also increased ferroptosis susceptibility. Clinical analyses further showed that elevated expression of *SELENOP* and *CCDC152* was associated with poor prognosis in astrocytoma, IDH-mutant. Together, these findings identify the CCDC152– SELENOP–LRP8 axis as a tumor-adapted selenium utilization system that couples extracellular selenium transport to intracellular redox homeostasis, revealing selenium utilization as a redox vulnerability that may be exploited by ferroptosis-inducing therapies.

## Highlights

• GBM cells establish a SELENOP–LRP8 cycle that reutilizes extracellular selenium.
• CCDC152 coordinates SELENOP production, LRP8 expression, and selenoprotein homeostasis.
• CCDC152 depletion lowers GPX1/GPX4 and increases ferroptosis susceptibility.
• The CCDC152– SELENOP–LRP8 axis represents a redox vulnerability in GBM.

## 1 Introduction

Glioblastoma, IDH-wildtype (GBM; CNS WHO grade 4), is the most aggressive primary malignant tumor of the adult central nervous system [1]. Its highly infiltrative nature makes complete surgical resection difficult, and despite radiotherapy and temozolomide chemotherapy, median overall survival remains approximately 15 months [1], [2]. GBM cells exhibit marked cellular heterogeneity and metabolic plasticity, enabling them to survive under hypoxia, oxidative stress, and nutrient limitation [1], [2], [3]. Ferroptosis, an iron-dependent form of regulated cell death driven by lipid peroxidation, has recently emerged as a potential vulnerability in GBM and may contribute to the antitumor effects of temozolomide [4], [5]. However, the mechanisms by which GBM cells acquire and retain essential trace elements required for redox homeostasis, ferroptosis resistance, and sustained proliferation remain poorly understood.

Selenium is an essential trace element incorporated into selenoproteins as the amino acid selenocysteine [6]. Selenoproteins, including glutathione peroxidases (GPXs) and thioredoxin reductases, play central roles in antioxidant defense, lipid peroxide metabolism, and cellular redox regulation [6], [7]. Because selenium is highly reactive and potentially toxic, its systemic transport is tightly regulated. Selenoprotein P (SELENOP), encoded by *SELENOP*, is the major extracellular selenium transport protein and contains multiple selenocysteine residues that enable efficient and controlled delivery of selenium to target tissues [8], [9]. SELENOP is synthesized predominantly in the liver and secreted into the circulation, where it transports selenium through receptor-mediated uptake. Members of the low-density lipoprotein receptor-related protein (LRP) family, including LRP8 (ApoER2), LRP2 (megalin), and LRP1, have been identified as SELENOP receptors with distinct tissue distributions [8], [9], [10]. Among these receptors, LRP8 is the principal mediator of selenium delivery to the brain and testis [11], [12]. Consistent with this role, *Selenop*- and *Lrp8*-deficient mice exhibit remarkably similar phenotypes, including reduced selenium levels in the brain and testis, neurological dysfunction, and impaired spermatogenesis [11]. Although the liver is the major source of circulating SELENOP, several extrahepatic tissues, including the brain, also express SELENOP, suggesting that selenium transport is regulated not only systemically but also within local tissue microenvironments [11], [12], [13], [14]. The importance of circulating SELENOP for selenium delivery to the brain was demonstrated in Selenop-knockout mice expressing a liver-specific human *SELENOP* transgene, which restored selenium transport to the brain and other selenium-dependent tissues [13], [14]. This finding provides a physiological basis for efficient SELENOP-mediated selenium delivery in the brain and supports the concept of a tumor-associated SELENOP cycle.

The coordinated processes of local SELENOP production, secretion, receptor-mediated uptake, and reutilization of SELENOP-derived selenium for intracellular selenoprotein synthesis constitute a functional SELENOP cycle [13], [14], [15]. Unlike the endocrine delivery of liver-derived SELENOP through the circulation, this local autocrine and paracrine cycle enables tissues to retain and redistribute selenium in a controlled form [11], [13], [14], [15]. The SELENOP cycle is physiologically important in the central nervous system: whole-body *Selenop* deficiency markedly reduces brain selenium, whereas liver-specific *Selenop* deletion does not, indicating a critical role for locally produced SELENOP in brain selenium retention [13], [14], [15], [16]. We previously demonstrated that *SELENOP* expression is elevated in GBM and that SELENOP depletion decreases GPX1 and GPX4 protein levels and enhances ferroptosis sensitivity, which is rescued by selenium supplementation [15]. Because GPX4 is a central suppressor of phospholipid peroxidation and ferroptosis, these findings led us to propose that GBM cells exploit a local selenium utilization system to preserve selenium availability and ferroptosis resistance [17], [18]. However, the receptor-dependent machinery, proliferative consequences, and upstream regulatory mechanisms of this tumor-associated SELENOP cycle remained undefined.

Recent studies have shown that the SELENOP–LRP8 pathway promotes glioma progression in addition to its established role in selenium transport [19]. SELENOP binds LRP8 and activates Wnt/β-catenin signaling, thereby enhancing glioma cell proliferation, migration, invasion, and tumor growth, indicating that LRP8 also functions as a signaling receptor in malignant glioma [19], [20]. However, how this pathway is established and maintained within tumor cells remains unclear. In particular, it is unknown whether highly proliferative GBM cells coordinately integrate SELENOP production and secretion, LRP8-mediated uptake, and intracellular selenium utilization into a tumor-associated SELENOP cycle. Moreover, despite the existence of multiple LRP8 splice variants and a conserved intracellular signaling domain, the structural determinants of its growth-promoting activity remain undefined [13], [21]. The upstream mechanisms coordinating SELENOP production, LRP8 expression, and selenium-dependent cellular functions also remain to be elucidated.

We previously identified *CCDC152* as an endogenous antisense lncRNA located at the *SELENOP* locus, with sequence complementarity to the 3′UTR/SECIS region of *SELENOP* mRNA [22]. *CCDC152* directly interacts with *SELENOP* mRNA and suppresses its translation by interfering with SBP2 binding and ribosome association, without affecting *SELENOP* mRNA stability or subcellular distribution [22]. We therefore designated this transcript lncRNA inhibitor of SELENOP translation (L-IST). Epigallocatechin gallate (EGCg) was previously identified as an inducer of *CCDC152/L-IST* expression, whereas its endogenous promoter architecture and detailed post-transcriptional processing, including splicing and polyadenylation, remain incompletely characterized [22]. In hepatocyte-derived cells, *CCDC152* functions as a negative regulator of SELENOP production. Unexpectedly, however, *Ccdc152* full-locus knockout mice exhibited little change in hepatic or circulating SELENOP levels, testicular selenoprotein expression, or systemic selenium homeostasis [23]. Instead, *Ccdc152* deficiency primarily affected genes involved in cytoskeletal organization and reproduction and produced modest defects in testicular architecture and sperm morphology [23]. These findings suggest that *CCDC152* is not a universal regulator of SELENOP expression and may have different roles depending on the cell type or tissue.

In the present study, we identified a tumor-associated SELENOP cycle in GBM in which tumor-derived SELENOP is secreted, recaptured through LRP8, and reutilized to sustain intracellular selenoprotein abundance and proliferation. We further identified *CCDC152* as an upstream coordinator of SELENOP production, LRP8 expression, and selenoprotein homeostasis. Together, these findings define a tumor-adapted selenium utilization pathway that links extracellular selenium transport to intracellular redox homeostasis and ferroptosis susceptibility. We additionally examined the clinical relevance of *SELENOP* and *CCDC152* expression across molecularly classified adult-type diffuse gliomas.

## 2 Methods

### 2.1 Cell culture

Human cancer cell lines T98G, YKG1, A172, U87, and 1321N1 were obtained from the Japanese Collection of Research Bioresources Cell Bank. 1321N1 was included as an astrocytoma-derived, low-SELENOP-expressing reference cell line for comparison with GBM-derived cell lines. T98G and 1321N1 cells were maintained in RPMI 1640 medium, whereas YKG1, A172, and U87 cells were maintained in high-glucose Dulbecco's modified Eagle's medium. All culture media were supplemented with 10% fetal bovine serum and 1% penicillin–streptomycin, and cells were maintained at 37°C in a humidified atmosphere containing 5% CO_2_.

Three patient-derived primary glioblastoma cell lines (PD-GBM1, PD-GBM2, and PD-GBM3) were established from surgically resected glioblastoma, IDH-wildtype (CNS WHO grade 4), specimens obtained at Tohoku University Hospital. Clinicopathological characteristics of the donor tumors are summarized in Supplementary Table S1. PD-GBM1 was derived from an MGMT promoter-unmethylated tumor, whereas PD-GBM2 and PD-GBM3 were established from MGMT promoter-methylated tumors, respectively. The methylation status of the MGMT gene promoter was assessed as previously described [24]. Briefly, DNA was extracted from the specimens using QIAamp DNA Mini Kit (Qiagen, Valencia, USA). Methylation status was assessed using multiplex ligation-dependent probe amplification with the SALSA MLPA kit (ME012 MGMT-IDH-TERT) in accordance with the manufacturer's protocol (MRC Holland, Amsterdam, the Netherland). Cells were maintained in Eagle's minimum essential medium supplemented with 10% fetal bovine serum and 1% penicillin–streptomycin. The establishment and experimental use of patient-derived cells were approved by the Ethics Committee of the Tohoku University Graduate School of Medicine (Approval No. 2023-1-321), and written informed consent was obtained from all patients. Cells were maintained in 10-cm culture dishes and passaged at approximately 80% confluence. For passaging and experimental use, cells were washed with phosphate-buffered saline, detached using 0.25% trypsin, collected by centrifugation, resuspended in the corresponding culture medium, and counted using a hemocytometer. For selenium supplementation experiments, sodium selenite (Na_2_SeO_3_; Sigma-Aldrich) was added to the culture medium at a final concentration of 100 nM. The duration of selenium supplementation was determined according to each experiment; cells were cultured for 48 h for the comparative *SELENOP* expression and secreted SELENOP analyses shown in Fig. 1A and B, whereas other experiments were performed for the periods indicated in the corresponding figure legends. For proliferation assays, cells were cultured in the presence or absence of 100 nM sodium selenite for the indicated experimental period.

**Fig. 1:**
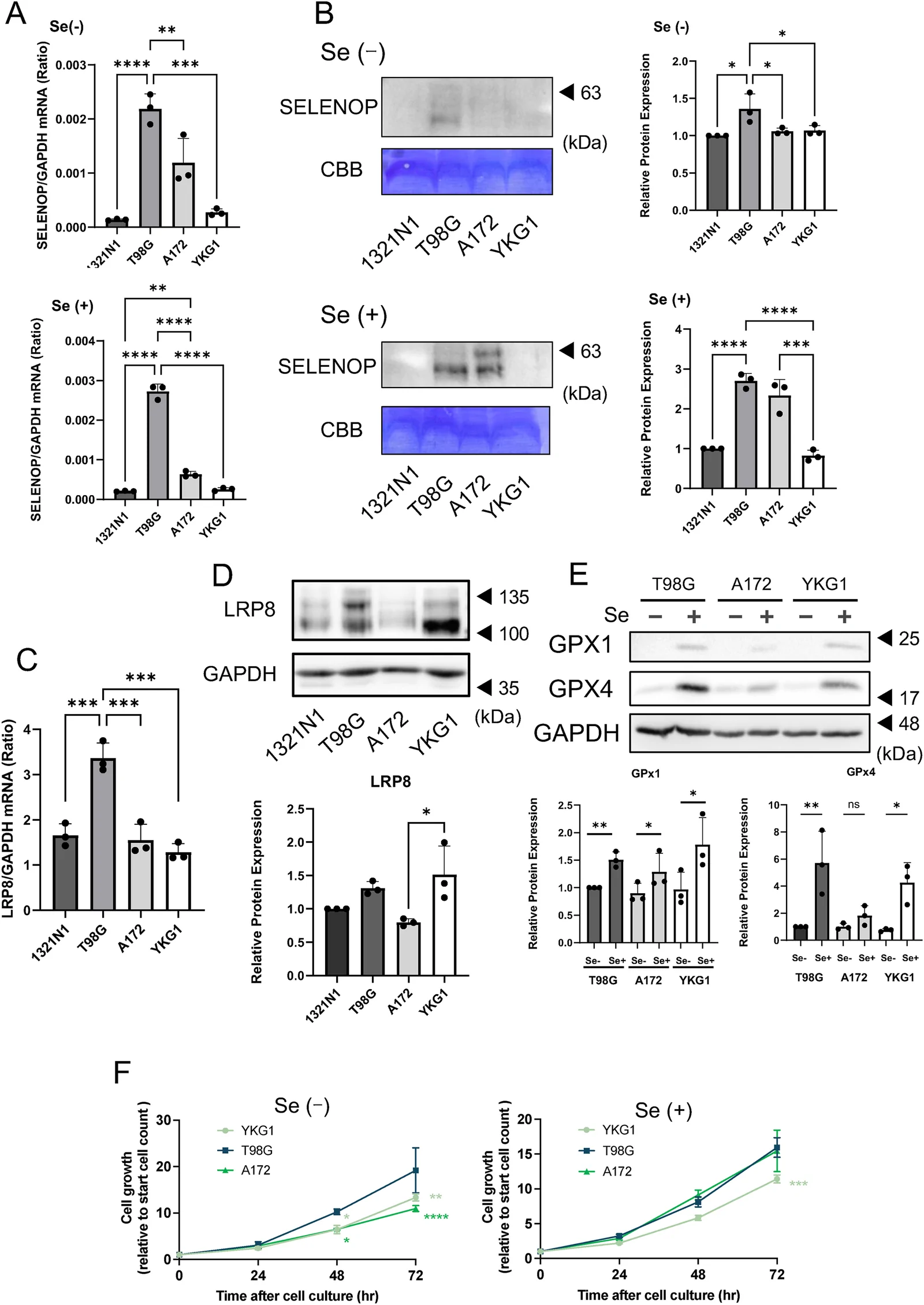
**Identification of a GBM model exhibiting robust SELENOP-cycle activity.** **A**, *SELENOP* mRNA expression in the astrocytoma-derived reference cell line 1321N1 and the GBM cell lines T98G, A172, and YKG1 cells cultured in the absence or presence of 100 nM sodium selenite for 48 h. **B**, Immunoblot analysis of SELENOP protein secreted into conditioned medium from 1321N1 and GBM cell lines cultured in the absence or presence of 100 nM sodium selenite for 48 h. Coomassie Brilliant Blue (CBB) staining is shown as a loading control. **C**, *LRP8* mRNA expression in glioma cell lines. **D**, Immunoblot analysis of LRP8 protein expression in glioma cell lines. **E**, Immunoblot analysis of intracellular GPX1 and GPX4 protein expression in GBM cell lines cultured in the absence or presence of 100 nM sodium selenite. GAPDH was used as a loading control. **F**, Cell proliferation of GBM cell lines cultured with or without 100 nM sodium selenite. Data are presented as mean ± SD from at least three independent experiments. Statistical significance was determined using one-way ANOVA followed by Tukey's multiple-comparison test (A, B, C, D, E), or two-way ANOVA followed by Tukey's multiple-comparison test (F), as appropriate. *P < 0.05, **P < 0.01, ***P < 0.001, and ****P < 0.0001.

### 2.2 Quantitative real-time PCR

Total RNA was isolated using ISOGEN II (Nippon Gene, Tokyo, Japan) according to the manufacturer's instructions. RNA concentration was determined using a NanoDrop spectrophotometer (Thermo Fisher Scientific). First-strand cDNA was synthesized using the PrimeScript RT Reagent Kit (Takara Bio, Shiga, Japan). Quantitative real-time PCR was performed using Power SYBR Green PCR Master Mix (Thermo Fisher Scientific) on a CFX Connect Real-Time PCR Detection System (Bio-Rad, Hercules, CA, USA). Relative mRNA expression levels were normalized to GAPDH. Primer sequences are listed in Supplementary Table S2.

### 2.3 Immunoblot analysis

Whole-cell lysates were prepared using RIPA buffer containing 50 mM Tris–HCl (pH 7.6), 150 mM NaCl, 1% Nonidet P-40 Substitute, 0.5% sodium deoxycholate, and 0.1% SDS. Protein concentrations were determined using the DC Protein Assay (Bio-Rad) according to the manufacturer's instructions. Equal amounts of protein were mixed with sample loading buffer, denatured at 95°C for 10 min, separated by SDS–polyacrylamide gel electrophoresis, and transferred onto polyvinylidene difluoride membranes. Membranes were blocked with 5% skim milk in Tris-buffered saline containing 0.1% Tween-20 (TBS-T) for 1 h at room temperature and then incubated with the indicated primary antibodies overnight at 4°C. After washing with TBS-T, membranes were incubated with horseradish peroxidase-conjugated secondary antibodies for 1 h at room temperature. Immunoreactive bands were detected using ImmunoStar LD chemiluminescent substrate (FUJIFILM Wako Pure Chemical Corporation, Osaka, Japan) and visualized using a Luminograph imaging system (ATTO, Tokyo, Japan). Antibodies used in this study are listed in Supplementary Table S3.

For analysis of secreted SELENOP, conditioned medium was collected and subjected to nickel-affinity bead enrichment as described below. The resulting eluates were analyzed by SDS–PAGE and immunoblotting. Equal loading and overall protein recovery were assessed by Coomassie Brilliant Blue (CBB) staining.

For immunoblot analyses, band intensities were quantified from three independent experiments unless otherwise specified in the figure legends. Intracellular protein levels were normalized to the corresponding loading control, such as GAPDH, whereas secreted SELENOP levels were normalized to the corresponding CBB staining, as appropriate. Quantitative data are presented as mean ± SD, and statistical analyses were performed as described in the Statistical analysis section.

### 2.4 Enrichment of secreted SELENOP from conditioned medium

To enrich SELENOP secreted into the culture medium, cells were seeded in 6-well plates at a density of 2.0 × 10^5^ cells in 2 mL of medium per well and cultured for 48 h. The medium was then replaced with 1 mL of fresh medium, and conditioned medium was collected after an additional 24 h. Cellular debris was removed by centrifugation. Secreted SELENOP was enriched from the conditioned medium using nickel-affinity beads. Briefly, 20 μL of well-suspended Ni beads was used per 1 mL of conditioned medium. The beads were collected by centrifugation at 500 × g for 5 min, equilibrated with ten bead volumes of equilibration buffer, and centrifuged again at 500 × g for 5 min. The conditioned medium was added to the equilibrated beads and incubated for 60 min at room temperature with end-over-end rotation. The beads were subsequently collected by centrifugation at 500 × g for 5 min and washed with washing buffer. Bound proteins were eluted by adding an equal volume of elution buffer and incubating the beads for 10 min at room temperature with end-over-end rotation. After centrifugation at 500 × g for 5 min, the supernatant containing the eluted proteins was transferred to a new tube. The elution procedure was repeated three times, and the resulting eluates were used for immunoblot analysis of secreted SELENOP [25].

### 2.5 Cell growth assay

Based on the cell counts, cells were seeded at the indicated densities for each experiment. For cell growth assays, cells were seeded into 6-well plates at a density of 7.0 × 10^4^ cells in 2 mL of culture medium per well. Cells were cultured for the indicated period under the indicated experimental conditions and then harvested for cell counting.

For selenium supplementation experiments, purified human SELENOP was added at the time of cell seeding at the indicated concentrations with or without antibodies, and cell numbers were determined after 72 h. For inhibitor experiments, cells were treated with the indicated concentrations of inhibitor at the time of seeding, and viable cell numbers were determined after 72 h using trypan blue exclusion and a hemocytometer.

### 2.6 siRNA-mediated knockdown

Gene-specific siRNAs targeting *SELENOP*, *LRP8*, or *CCDC152*, together with a non-targeting control siRNA, were purchased from Sigma-Aldrich (St. Louis, MO, USA). Cells were transfected with siRNAs using Lipofectamine RNAiMAX Transfection Reagent (Thermo Fisher Scientific, Waltham, MA, USA) according to the manufacturer's instructions (10 nM siRNA and 1 μL Lipofectamine RNAiMAX per mL culture medium). Cells were harvested 48 h after transfection for RNA extraction, immunoblot analysis, or cell growth assays, as indicated. Knockdown efficiency was confirmed by quantitative real-time PCR and, where applicable, by immunoblot analysis. The siRNA sequences are listed in Supplementary Table S4. For *CCDC152*, two independent siRNAs targeting distinct regions of the transcript were used to induce RNAi-mediated depletion of *CCDC152*, which was confirmed by quantitative real-time PCR.

### 2.7 Generation of SELENOP-overexpressing YKG1 cells

*SELENOP*-overexpressing YKG1 cells were generated by lentiviral transduction. The *SELENOP* transgene contained the full coding sequence together with the 3′UTR including the SECIS elements required for selenocysteine incorporation. Lentiviral particles were produced in HEK293T cells by co-transfection of a *SELENOP* transfer vector with the packaging plasmid psPAX2 and the envelope plasmid pMD2.G using polyethylenimine. Viral supernatants were collected, filtered, and used to transduce YKG1 cells. At 48 h after transduction, cells were selected with puromycin (1.5 μg/mL) for an additional 48 h. SELENOP overexpression was confirmed by quantitative real-time PCR and immunoblot analysis of secreted SELENOP; however, the number of Sec residues incorporated per SELENOP molecule was not directly determined.

### 2.8 Ferroptosis sensitivity assay

T98G cells were transfected with a non-targeting control siRNA or two independent siRNAs targeting *CCDC152*. At 24 h after transfection, cells were treated with RSL3 (10 nM) or erastin (1 μM) in the absence or presence of deferoxamine (DFO; 50 μM) for an additional 24 h. Cell viability was assessed using alamarBlue Cell Viability Reagent (Thermo Fisher Scientific). The culture medium was replaced with medium containing 10% alamarBlue reagent, and cells were incubated for 2 h before fluorescence was measured at an excitation wavelength of 544 nm and an emission wavelength of 585 nm using a SpectraMax iD5 microplate reader (Molecular Devices).

### 2.9 Preparation of purified human SELENOP protein

Purified human SELENOP was prepared from pooled human plasma obtained from multiple blood donors as previously described [26]. Briefly, pooled human plasma was sequentially subjected to heparin-affinity and anion-exchange chromatography. SELENOP-containing fractions were further purified and concentrated using Ni-agarose affinity chromatography, followed by desalting with a PD-10 desalting column (Cytiva, Buckinghamshire, UK). Human frozen plasma was provided by the Japanese Red Cross Tohoku Block Blood Center under Institutional Review Board approval (No. 25J0012). Neither the selenium content of the pooled plasma nor the Sec occupancy of the purified SELENOP was directly determined. SELENOP concentration was determined by ELISA [25], and the presence of SELENOP fragments in the purified preparation was examined; however, the precise number of Sec residues incorporated per SELENOP molecule was not determined.

### 2.10 Cellular uptake of purified SELENOP

Purified human SELENOP was added to T98G cells at the indicated concentrations. After incubation for 3 h, cells were washed three times with ice-cold PBS to remove extracellular SELENOP before preparation of whole-cell lysates. Intracellular SELENOP uptake and LRP8 protein expression were subsequently analyzed by immunoblotting.

### 2.11 Effect of anti-human SELENOP antibodies on cell growth

Monoclonal antibodies recognizing the C-terminal region (AA3) or histidine-rich domain (BD3) of human SELENOP were prepared as previously described [25]. For cell growth assays, T98G cells were seeded into 6-well plates at a density of 7.0 × 10^4^ cells in 2 mL of culture medium per well. AA3 or BD3 antibody was added at the time of cell seeding at a final concentration of 20 μg/mL in the presence of 100 nM sodium selenite. Viable cell numbers were determined after 72 h using trypan blue exclusion and a hemocytometer.

### 2.12 RNA sequencing and gene set enrichment analysis

Total RNA was isolated from control and LRP8-knockdown T98G cells and submitted to GenomeLead Co., Ltd. for RNA sequencing. Raw sequencing reads were subjected to quality control using FastQC and adapter trimming using Trimmomatic. Ribosomal RNA-derived reads were removed using Bowtie2, and the remaining reads were aligned to the human reference genome using STAR. Gene-level read counts were obtained using featureCounts, and differential expression analysis was performed using DESeq2 with the Wald test. Functional pathway enrichment analysis was performed using Metascape with KEGG and Reactome gene-set libraries.

### 2.13 Construction of LRP8 splice variants

Expression plasmids encoding full-length human LRP8 and LRP8 splice variants lacking the intracellular domain were generated by PCR-based cloning into the pcDNA3.1 expression vector and verified by Sanger sequencing. Plasmids were transiently transfected into T98G cells using Lipofectamine 2000 Transfection Reagent (Thermo Fisher Scientific, Waltham, MA, USA) according to the manufacturer's instructions (1 μg plasmid DNA and 1 μL Lipofectamine 2000 per mL culture medium). Cells were harvested 24 h after transfection for subsequent analyses, including immunoblot analysis and cell growth assays.

### 2.14 Animal housing and ethical approval

Animals were maintained under specific pathogen-free conditions in a temperature- and humidity-controlled facility (22 ± 1°C, 55% humidity) under a 12-h light/dark cycle, with free access to standard laboratory chow and water. All animal experiments were approved by the Institutional Animal Care and Use Committee of Tohoku University (approval No. 2022-005-03) and conducted in accordance with institutional guidelines.

### 2.15 Subcutaneous xenograft model

Male BALB/cAJclnu/nu mice (7 weeks old; Japan CLEA, Shizuoka, Japan) were used for the subcutaneous xenograft experiments. T98G cells transfected with a non-targeting control siRNA or siRNAs targeting LRP8 or CCDC152 were harvested and resuspended in sterile PBS. A total of 2 × 10^6^ cells in 200 μL PBS were injected subcutaneously into the inguinal region of nude mice. Tumor dimensions were measured periodically using calipers, and tumor volume was calculated as length × width^2^/2.

### 2.16 Orthotopic xenograft model

U87 cells stably expressing firefly luciferase (U-87 MG-Luc2, HTB-14-LUC2, ATCC) were transfected with a non-targeting control siRNA (siCON) or an siRNA targeting LRP8 (siLRP8) 48 h before implantation. Cells were harvested, washed twice with ice-cold PBS, and resuspended in PBS at a concentration of 1.0 × 10^5^ cells/μL. Throughout the preparation, cell suspensions were maintained on ice. Male F344/NJcl-rnu/rnu rats (7 weeks old; Japan CLEA, Shizuoka, Japan) were used for the orthotopic xenograft experiments. Rats were anesthetized and positioned in a stereotaxic apparatus. A total of 2.0 × 10^5^ cells in 2 μL of PBS was stereotactically injected into the right striatum using a Hamilton syringe. The injection site was located 0.5 mm anterior and 3 mm lateral to the bregma, and the needle was advanced to a depth of 5 mm from the brain surface and then withdrawn by 0.5 mm before injection. The cell suspension was injected over 4 min, and the needle was left in place for an additional 2 min before being slowly withdrawn. All animal experiments were approved by the Animal Care and Use Committee of Tohoku University (approval No. 2022-005-03) and were performed in accordance with institutional guidelines.

### 2.17 Bioluminescence imaging

Bioluminescence imaging was performed 32 days after intracranial implantation. Rats received an intraperitoneal injection of CycLuc1 (1.4 mg/kg), and images were acquired 10 min after administration using an IVIS Spectrum imaging system with an open emission filter. Bioluminescence images were generated and analyzed using Living Image software version 4.3 (PerkinElmer). Photon flux within a defined region of interest encompassing the intracranial tumor was quantified using identical acquisition and analysis settings for all animals.

### 2.18 Construction of CCDC152 expression vector

Full-length human *CCDC152* cDNA was amplified by PCR and cloned into the pcDNA3.1 mammalian expression vector. The resulting construct was verified by Sanger sequencing before use. T98G or YKG1 cells were transiently transfected with the *CCDC152* expression vector or the corresponding empty vector using Lipofectamine 2000 Transfection Reagent (Thermo Fisher Scientific, Waltham, MA, USA) according to the manufacturer's instructions (1 μg plasmid DNA and 1 μL Lipofectamine 2000 per mL culture medium). Cells were harvested 24 h after transfection for subsequent analyses, including quantitative real-time PCR, immunoblot analysis, and cell growth assays.

To assess the coding potential of *CCDC152*, T98G cells were transiently transfected with an HA-tagged *CCDC152* expression construct, an HA-tagged *iNOS* expression construct as a positive control, or the corresponding empty vector using Lipofectamine 2000 under the conditions described above. Cells were harvested 24 h after transfection. *CCDC152* and *iNOS* transcript expression was assessed by quantitative real-time PCR, and expression of HA-tagged proteins was examined by immunoblotting using an anti-HA antibody.

### 2.19 Human glioma datasets

RNA-sequencing and clinical data from The Cancer Genome Atlas (TCGA) and the Genotype-Tissue Expression (GTEx) project, uniformly processed using the Toil pipeline, were obtained from the UCSC Xena platform and analyzed using R version 4.5.2. Gliomas were classified according to IDH mutation and 1p/19q-codeletion status. Differences in gene expression between each tumor group and normal brain tissues were assessed using two-sided Welch's t-tests with Holm correction for multiple comparisons. For overall survival analyses, patients were stratified into high- and low-expression groups using the median expression level as the cutoff, and survival curves were compared using the log-rank test.

### 2.20 Human glioma specimens

Formalin-fixed, paraffin-embedded human glioma specimens were obtained from patients who underwent surgical resection at Tohoku University Hospital. For longitudinal analysis, paired primary and recurrent astrocytoma, IDH-mutant, specimens obtained from the same patients were examined. Histopathological diagnosis and tumor grading were established according to the 2021 WHO Classification of Tumors of the Central Nervous System, 5th edition by board-certified neuropathologists. The study was approved by the Institutional Review Board of Tohoku University (Approval No. 2026-1-309), and written informed consent was obtained from all patients in accordance with the Declaration of Helsinki.

### 2.21 Immunohistochemistry

Formalin-fixed, paraffin-embedded human glioma tissue sections (4 μm thick) were deparaffinized in xylene, rehydrated through a graded ethanol series, and washed with distilled water. Endogenous peroxidase activity was quenched with 0.3% hydrogen peroxide in methanol. Antigen retrieval was performed in citrate buffer (pH 6.0) using an autoclave (121°C for 5 min). After washing with distilled water and 0.01 M PBS, sections were blocked using Histofine blocking solution (Nichirei Biosciences) for 30 min at room temperature. Sections were then incubated overnight at 4°C with the rat monoclonal anti-human SELENOP antibody BD1 (42.5 μg/mL) diluted in antibody diluent (0.5% bovine serum albumin in PBS containing 0.05% sodium azide) [25]. After washing with PBS, sections were incubated with biotinylated anti-rat IgG (20 μg/mL) for 30 min at room temperature, followed by horseradish peroxidase-conjugated streptavidin (Histofine Kit, Nichirei Biosciences) for 30 min. Immunoreactivity was visualized with 3,3′-diaminobenzidine (DAB), and sections were counterstained with hematoxylin before dehydration, clearing, and mounting.

SELENOP immunoreactivity was independently evaluated by two investigators blinded to the clinical information. Human liver tissue, which constitutively expresses SELENOP, was included as a positive control. Tumor staining intensity was classified relative to liver tissue as negative, weakly positive, positive (comparable to liver), or strongly positive (stronger than liver). Discrepant evaluations were resolved by consensus.

### 2.22 Survival analysis

Overall survival was defined as the interval between the date of surgery and death from any cause or the date of the last follow-up. Survival curves were generated using the Kaplan–Meier method and compared using the log-rank test. For immunohistochemistry-based survival analysis, cases with negative or weakly positive SELENOP staining were combined as the low-expression group, whereas positive and strongly positive cases were analyzed as the high-expression group because the former two categories exhibited comparable clinical outcomes.

### 2.23 Statistical analysis

Statistical analyses were performed using GraphPad Prism 10 (GraphPad Software, Boston, MA, USA), unless otherwise indicated. Data are presented as the mean ± SD from the number of independent experiments specified in the figure legends. Comparisons between two groups were performed using an unpaired two-tailed Student's *t*-test or Welch's *t*-test, as appropriate. Comparisons among three or more groups were performed using one-way analysis of variance (ANOVA), followed by Tukey's or Dunnett's multiple-comparison test, as indicated. Experiments involving two independent variables were analyzed using two-way ANOVA followed by Tukey's multiple-comparison test. Kaplan–Meier survival curves were compared using the log-rank test. All statistical tests were two-sided, and *P* < 0.05 was considered statistically significant.

## 3 Results

### 3.1 Identification of a GBM model exhibiting robust SELENOP-cycle activity

To investigate the molecular basis of the SELENOP cycle in GBM, we first sought to identify GBM cell lines exhibiting high SELENOP-cycle activity. Among the glioma cell lines examined, including the astrocytoma-derived cell line 1321N1 and three established GBM cell lines, T98G cells exhibited the highest *SELENOP* mRNA expression irrespective of selenium supplementation (Fig. 1A). Consistent with these transcript levels, secreted SELENOP protein was readily detected in conditioned medium from T98G cells and became detectable in A172 cells following selenium supplementation, whereas it was not detected in 1321N1 and YKG1 cells (Fig. 1B), indicating robust SELENOP production and secretion. Consistent with high *SELENOP* expression, LRP8 expression was also high in T98G cells at both the mRNA and protein levels (Fig. 1C and D). In contrast, expression of the other reported SELENOP receptors, *LRP1* and *LRP2*, was not preferentially elevated in T98G cells (Supplementary Fig. S1). These findings identified T98G cells as a representative GBM model with robust SELENOP-cycle activity. We next examined intracellular selenoprotein expression. Under basal culture conditions, GPX expression showed only modest differences among the GBM cell lines. In contrast, selenium supplementation preferentially increased GPX4 expression in T98G cells, resulting in the highest GPX levels among the cell lines examined (Fig. 1E). Although T98G cells exhibited the highest proliferative capacity under basal conditions, selenium supplementation enhanced proliferation in all GBM cell lines, thereby reducing the differences in proliferation among the cell lines (Fig. 1F).

To confirm these findings in a clinically relevant model, we next examined patient-derived GBM (PD-GBM) cells. PD-GBM1 cells exhibited the highest *SELENOP* expression and also displayed the greatest proliferative capacity among the PD-GBM cell lines examined (Supplementary Fig. S2), supporting an association between elevated SELENOP expression and rapid GBM cell growth. Collectively, these findings identify T98G cells as a representative GBM model exhibiting robust SELENOP-cycle activity and suggest that activation of the SELENOP cycle is associated with a highly proliferative phenotype in GBM.

### 3.2 Tumor-derived SELENOP sustains GBM proliferation

To determine whether SELENOP directly contributes to GBM cell proliferation, we first suppressed endogenous *SELENOP* expression in T98G cells using three independent siRNAs. All siRNAs efficiently reduced *SELENOP* mRNA expression and SELENOP protein secretion into the culture medium (Fig. 2A and B). Under these conditions, cell proliferation was significantly reduced by all three siRNAs (Fig. 2C), demonstrating that endogenous SELENOP supports GBM cell proliferation. We next examined whether selenium supplementation could rescue the growth defect induced by *SELENOP* knockdown. Selenium supplementation partially restored proliferation following *SELENOP* knockdown, although siRNA #3 retained significant inhibitory activity after 72 h (Fig. 2D). These findings indicate that exogenous selenium partially rescues the growth defect caused by *SELENOP* depletion, potentially by increasing intracellular selenium availability and by enhancing residual SELENOP production and LRP8-mediated selenium uptake or signaling.

**Fig. 2:**
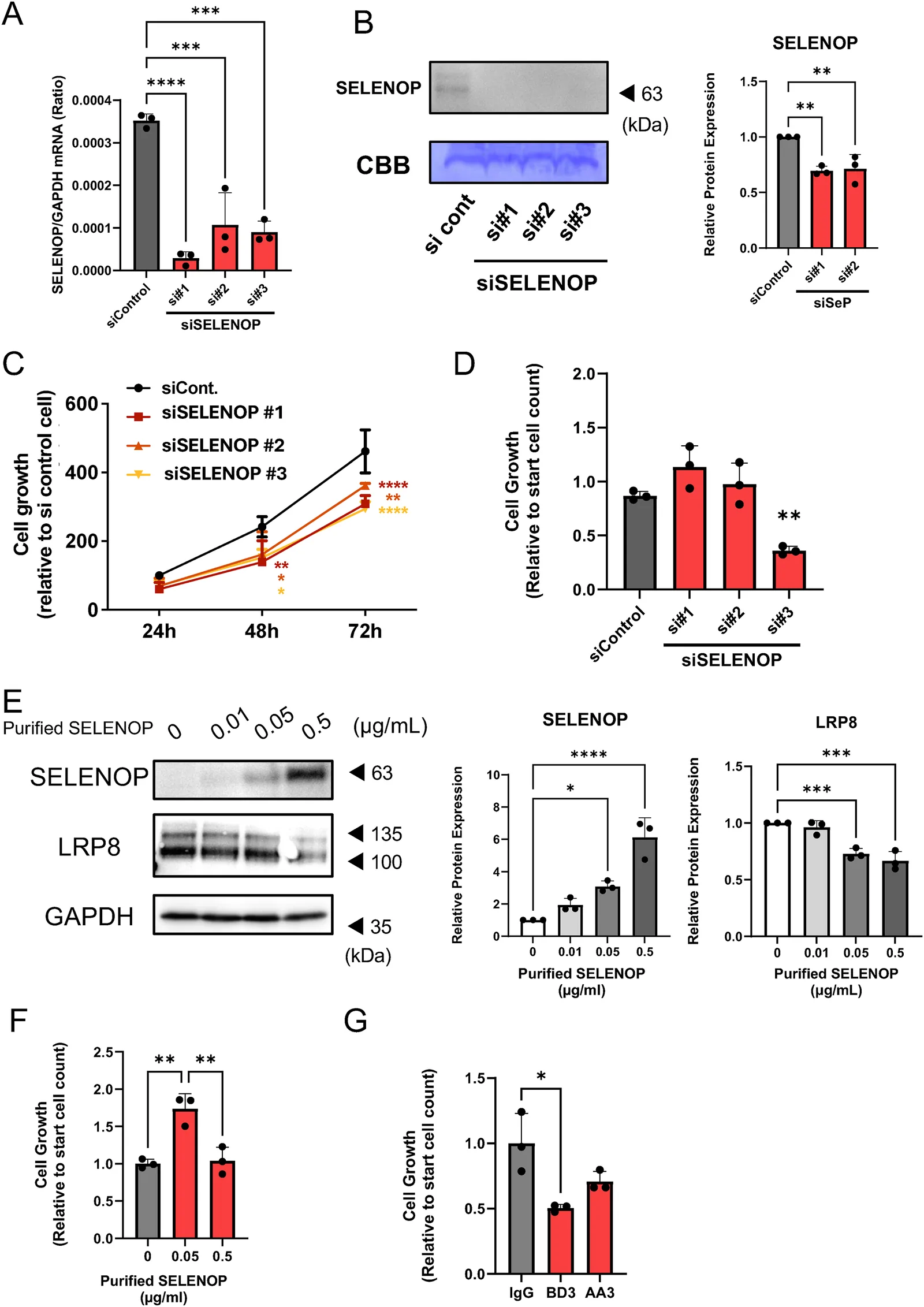
**Tumor-derived SELENOP promotes GBM proliferation through an autocrine SELENOP–LRP8 circuit.** **A**, Knockdown efficiency of three independent *SELENOP* siRNAs determined by quantitative PCR. **B**, Immunoblot analysis of SELENOP protein secreted into conditioned medium following *SELENOP* knockdown. CBB staining is shown as a loading control. **C**, Effect of *SELENOP* knockdown on T98G cell proliferation. **D**, Effect of supplementation with 100 nM sodium selenite on cell proliferation following *SELENOP* knockdown. **E**, Immunoblot analysis of intracellular uptake of purified human SELENOP and endogenous LRP8 expression following SELENOP treatment. GAPDH was used as a loading control. **F**, Effect of purified human SELENOP on T98G cell proliferation. Cells were treated with purified human SELENOP (0.01–0.5 μg/mL) for 72 h. **G**, Effect of anti-SELENOP monoclonal antibodies recognizing the C-terminal region (AA3) or histidine-rich domain (BD3) on T98G cell proliferation [27]. Data are presented as mean ± SD from three independent experiments. Statistical significance was determined using one-way ANOVA followed by Tukey's multiple-comparison test (A, B, D, E, F, G), or two-way ANOVA followed by Tukey's multiple-comparison test (C), as appropriate. *P < 0.05 and **P < 0.01.

To further determine whether extracellular SELENOP directly promotes GBM cell proliferation, purified human SELENOP protein was added to T98G cells. SELENOP was taken up into cells in a concentration-dependent manner, whereas treatment with the highest concentration (0.5 μg/mL) reduced LRP8 protein levels (Fig. 2E). Consistent with these observations, addition of 0.05 μg/mL SELENOP significantly promoted cell proliferation, whereas exposure to the highest concentration (0.5 μg/mL) was accompanied by reduced LRP8 protein expression (Fig. 2E and F). Likewise, forced SELENOP overexpression enhanced cell proliferation, indicating that modest increases in extracellular SELENOP are sufficient to stimulate GBM cell growth (Supplementary Fig. S3). Finally, we examined whether disruption of the SELENOP–LRP8 interaction affects cell proliferation. Treatment with anti-SELENOP monoclonal antibodies recognizing either the C-terminal region (AA3) or the histidine-rich domain (BD3) significantly inhibited T98G cell proliferation (Fig. 2G). Collectively, these findings support the concept that tumor-derived SELENOP functions as an autocrine growth factor sustaining GBM proliferation through LRP8-mediated uptake.

### 3.3 LRP8 promotes GBM proliferation through its intracellular domain

To determine how LRP8 mediates the growth-promoting activity of tumor-derived SELENOP, we first suppressed LRP8 expression in T98G cells using three independent siRNAs. Efficient knockdown of LRP8 was confirmed at both the mRNA and protein levels (Fig. 3A and B). LRP8 depletion was accompanied by reduced GPX1 and GPX4 expression at both the mRNA and protein levels, indicating that LRP8 contributes to intracellular selenium utilization and maintenance of selenoprotein homeostasis (Fig. 3B and Supplementary Fig. S4). To identify downstream pathways regulated by LRP8, transcriptome analysis was performed following *LRP8* knockdown. Pathway enrichment analysis revealed significant alterations in pathways associated with cell-cycle progression (Fig. 3C). Consistent with these transcriptional changes, *LRP8* knockdown significantly suppressed T98G cell proliferation, and this effect was reproduced using three independent *LRP8*-targeting siRNAs (Fig. 3D and Supplementary Fig. S5). Because LRP8 undergoes extensive alternative splicing, we next investigated which structural domain mediates its growth-promoting activity. Expression vectors encoding full-length LRP8 or representative splice variants were transiently introduced into T98G cells (Fig. 3E). Whereas full-length LRP8 significantly enhanced cell proliferation, splice variants lacking exon 18 failed to promote cell growth (Fig. 3F and G). These findings identify the intracellular domain encoded by exon 18 as an essential structural determinant underlying the growth-promoting activity of LRP8.

**Fig. 3:**
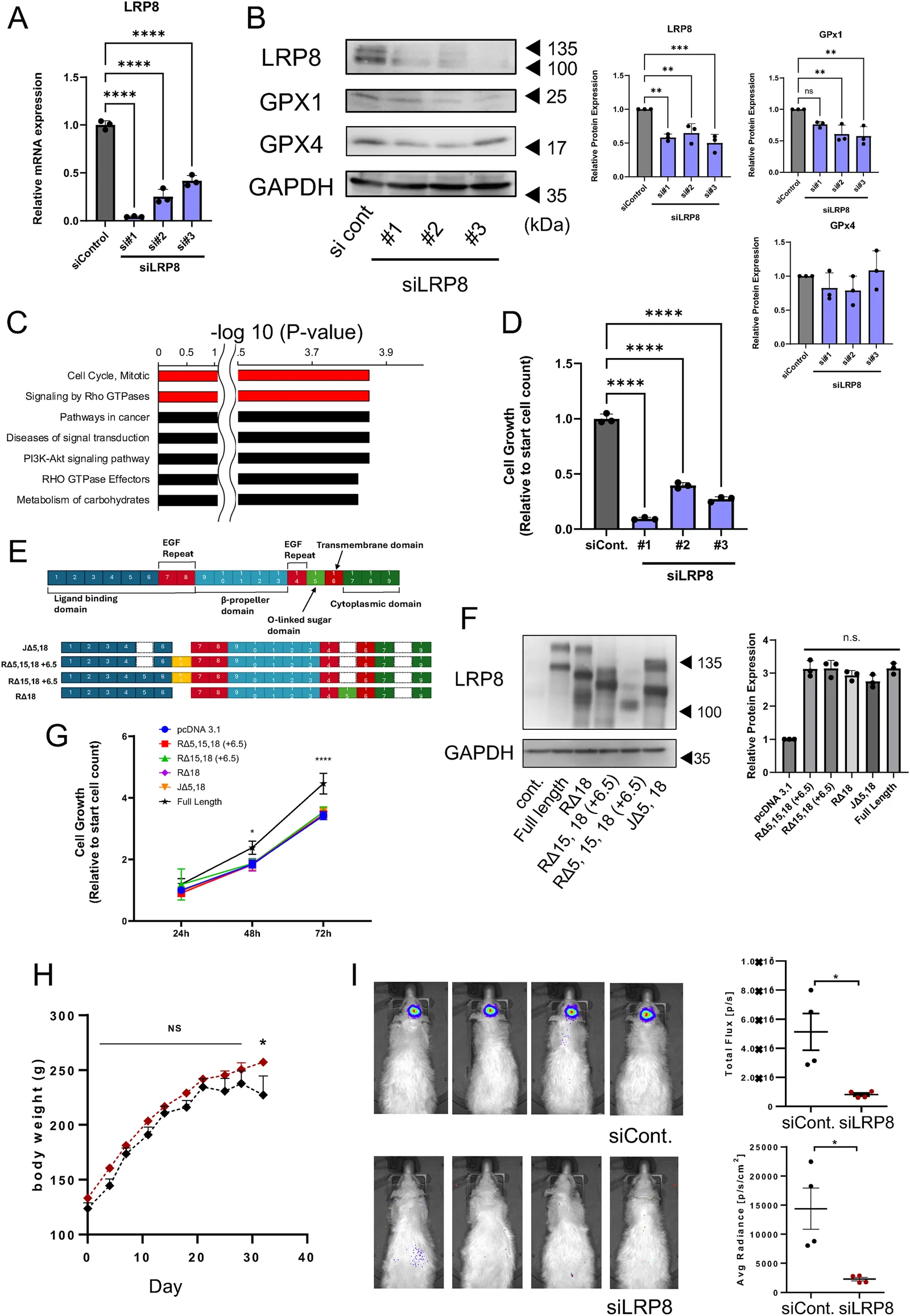
**LRP8 promotes GBM proliferation through its intracellular domain.** **A**, Knockdown efficiency of *LRP8* siRNAs determined by quantitative PCR. **B**, Immunoblot analysis of LRP8, GPX1, and GPX4 following *LRP8* knockdown. GAPDH was used as a loading control. **C**, Pathway enrichment analysis of RNA-sequencing data following *LRP8* depletion. **D**, Effect of *LRP8* knockdown on T98G cell proliferation. **E**, Schematic illustration of full-length *LRP8* and representative splice variants used in this study. **F**, Immunoblot analysis of LRP8 splice variants following transient transfection. GAPDH was used as a loading control. **G**, Effects of LRP8 splice variants on cell proliferation. **H**, Changes in body weight following intracranial implantation of control or *LRP8*-depleted U87 cells. **I**, Representative bioluminescence images and quantification of photon flux of intracranial xenografts established with control or *LRP8*-depleted U87 cells at day 32 after implantation. Data are presented as mean ± SD from three independent experiments unless otherwise indicated. Statistical significance was determined using one-way ANOVA followed by Tukey's multiple-comparison test (A, B, D, F), or two-way ANOVA followed by Tukey's multiple-comparison test (G, H), or Welch's *t*-test (I), as appropriate. *P < 0.05 and ****P < 0.0001.

Finally, we evaluated the contribution of LRP8 to GBM growth in vivo. *LRP8* siRNA-transfected T98G cells were implanted into nude mice, whereas *LRP8* siRNA-transfected firefly luciferase-expressing U87 GBM cells were stereotactically implanted into the rat brain using a stereotactic apparatus. *LRP8* knockdown significantly reduced tumor growth in the subcutaneous xenograft model (Supplementary Fig. S6A and S6B). Likewise, bioluminescence imaging demonstrated that LRP8-depleted cells displayed markedly impaired intracranial tumor growth in the rat brain, accompanied by attenuation of tumor-associated body-weight loss after implantation (Fig. 3H and I). Collectively, these findings identify the intracellular domain of LRP8 as a critical signaling determinant linking the SELENOP cycle to GBM proliferation and tumor growth.

### 3.4 CCDC152 positively regulates the SELENOP cycle in GBM

*CCDC152* is a lncRNA located antisense to the *SELENOP* locus [22], [23]. We previously identified *CCDC152* as a specific inhibitor of *SELENOP* translation in hepatocytes. We next investigated whether it contributes to the SELENOP cycle in GBM. Single-cell transcriptomic analysis of public glioma datasets showed that *CCDC152* expression increased with glioma malignancy (Fig. 4A and Supplementary Fig. S7). Further, *CCDC152* was highly expressed in glioma-derived cell lines, with the highest expression observed in T98G cells (Fig. 4B). These findings suggested that *CCDC152* may play a distinct role in malignant glioma compared with that previously observed in hepatocyte-derived cells.

**Fig. 4:**
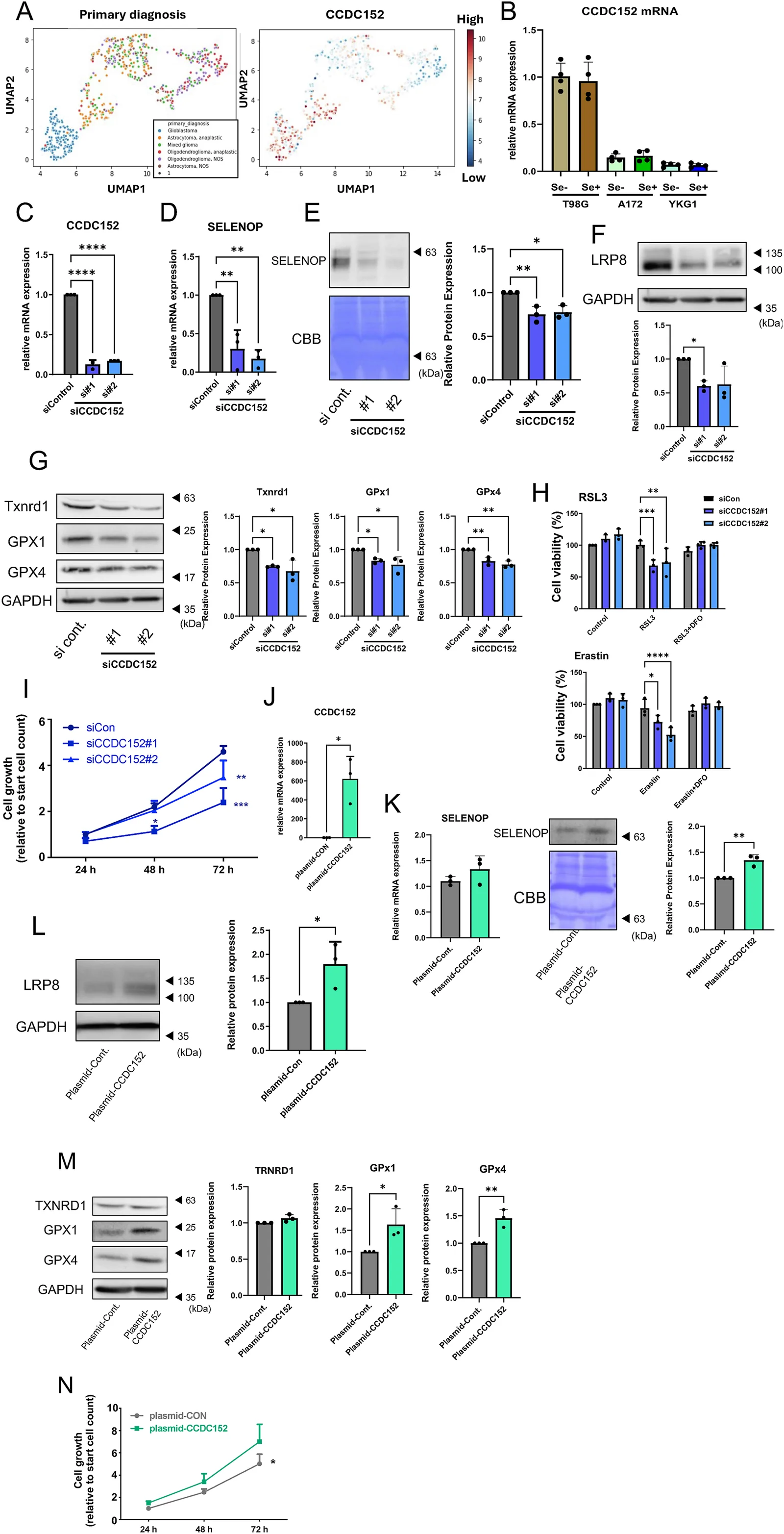
***CCDC152* positively coordinates the tumor-associated SELENOP cycle and ferroptosis in GBM.** **A**, Single-cell expression of *CCDC152* across human gliomas visualized by UMAP. Left, primary diagnosis labels are shown as originally annotated in the source dataset. Right, *CCDC152* expression levels, with red and blue indicating high and low expression, respectively. An enlarged version of Fig. 4A is provided in Supplementary Fig. S7 B, Basal expression of *CCDC152* in human GBM cell lines. Relative *CCDC1*52 mRNA expression in T98G, A172, and YKG1 cells cultured in the absence or presence of 100 nM sodium selenite for 24 h. Expression levels were normalized to *GAPDH*. **C**, Knockdown efficiency of two independent *CCDC152* siRNAs in T98G cells determined by quantitative PCR. **D**, *SELENOP* mRNA expression following *CCDC152* knockdown. **E**, Immunoblot analysis of secreted SELENOP in conditioned medium following *CCDC152* knockdown. CBB staining is shown as a loading control. **F**, *LRP8* mRNA expression and immunoblot analysis of LRP8 protein following *CCDC152* knockdown. **G**, Immunoblot analysis of intracellular selenoproteins following *CCDC152* knockdown. **H**, CCDC152 depletion increases ferroptosis sensitivity in GBM cells. Sensitivity of T98G cells to RSL3-and Erastin-induced ferroptosis was evaluated in the absence or presence of the ferroptosis inhibitor deferoxamine (DFO), and cell viability was determined after the indicated treatment period. I, Effect of *CCDC152* knockdown on T98G cell proliferation. **J**, Forced expression of *CCDC152* in YKG1 cells determined by quantitative PCR. **K**, *SELENOP* mRNA expression and immunoblot analysis of SELENOP protein secreted into conditioned medium following *CCDC152* overexpression. CBB staining is shown as a loading control. **L**, *LRP8* mRNA expression and immunoblot analysis of LRP8 protein following *CCDC152* overexpression. Data for SELENOP mRNA expression are presented as mean ± SD from three independent experiments (n = 3), and the immunoblot shown is representative of three independent experiments. **M**, Immunoblot analysis of intracellular GPX1, GPX4, and TXNRD1 following *CCDC152* overexpression. GAPDH was used as a loading control. **N**, Effect of *CCDC152* overexpression on YKG1 cell proliferation. Data are presented as mean ± SD from three independent experiments unless otherwise indicated. Statistical significance was determined using one-way ANOVA followed by Tukey's multiple-comparison test (C, D, E, F, G, H), or two-way ANOVA followed by Tukey's multiple-comparison test (I, N), or Welch's *t*-test (J, K, L, M), as appropriate. *P < 0.05, **P < 0.01, ***P < 0.001, and ****P < 0.0001.

We therefore depleted *CCDC152* in T98G cells using independent siRNAs. Efficient knockdown of *CCDC152* was confirmed by quantitative PCR (Fig. 4C). Unexpectedly, *CCDC152* depletion reduced *SELENOP* expression and SELENOP secretion into the culture medium (Fig. 4D and E). Thus, in contrast to its previously identified inhibitory effect on *SELENOP* translation in hepatocytes, endogenous *CCDC152* positively regulated SELENOP expression in GBM cells. We next examined whether *CCDC152* also affected other components of the SELENOP cycle. *CCDC152* knockdown also reduced the levels of LRP8, GPX1, and GPX4, indicating impaired intracellular selenoprotein homeostasis (Fig. 4F and G). Accordingly, *CCDC152*-depleted cells became more susceptible to ferroptosis (Fig. 4H). In addition, *CCDC152* knockdown significantly delayed cell proliferation (Fig. 4I). Collectively, these findings indicate that endogenous *CCDC152* supports both selenium utilization and GBM cell proliferation. Finally, consistent with the in vitro findings, knockdown of *CCDC152* also suppressed subcutaneous tumor growth in vivo (Supplementary Fig. S6C), supporting a role for *CCDC152* as a positive regulator of the SELENOP–LRP8 signaling axis in GBM. Conversely, we examined the effect of forced *CCDC152* expression in YKG1 cells, which exhibited minimal endogenous *CCDC152* expression. Successful overexpression of *CCDC152* slightly increased *SELENOP* mRNA expression and increased SELENOP protein levels in conditioned medium (Fig. 4J and K). *CCDC152* overexpression also increased LRP8 protein expression (Fig. 4L) and elevated the levels of intracellular selenoproteins, including GPX1 and GPX4 (Fig. 4M). Consistent with activation of these components of the SELENOP cycle, forced *CCDC152* expression significantly enhanced YKG1 cell proliferation (Fig. 4N). Finally, because *CCDC152* contains a predicted open reading frame, we examined whether its biological activity could be attributed to a translated protein. Although ectopic expression of an HA-tagged *CCDC152* construct resulted in robust *CCDC1*52 mRNA expression, no detectable HA-tagged protein was observed by immunoblotting. In contrast, an HA-tagged *iNOS* construct generated a readily detectable protein under identical experimental conditions (Supplementary Fig. S8). These findings support the interpretation that *CCDC152* functions primarily as a lncRNA under the present experimental conditions. Collectively, these findings identify *CCDC152* as an upstream regulator of the tumor-associated SELENOP cycle in GBM.

### 3.5 Clinical relevance of SELENOP and CCDC152 expression in molecularly classified diffuse gliomas

To investigate the clinical relevance of the tumor-associated SELENOP cycle, we examined the expression of *SELENOP* and *LRP8* in molecularly classified diffuse gliomas using uniformly processed TCGA and GTEx RNA-sequencing data obtained from the UCSC Xena platform. *SELENOP* expression was significantly higher in GBM, astrocytoma, IDH-mutant, oligodendroglioma, IDH-mutant and 1p/19q-codeleted, and IDH-wildtype diffuse glioma than in normal brain tissues (Fig. 5A). By contrast, *LRP8* expression was significantly increased in astrocytoma, oligodendroglioma, and IDH-wildtype diffuse glioma but not in GBM (Supplementary Fig. S9). We next examined the association between *SELENOP* expression and patient prognosis. Elevated *SELENOP* expression was significantly associated with shorter overall survival specifically in astrocytoma, whereas no significant association was observed in GBM, oligodendroglioma, or IDH-wildtype diffuse glioma (Fig. 5B). In contrast, *LRP8* expression was not significantly associated with overall survival in any molecular subtype (Supplementary Fig. S10). These findings indicate that, within the transcriptomic datasets analyzed, a significant association between *SELENOP* mRNA expression and overall survival was detected only in astrocytoma, IDH-mutant.

**Fig. 5:**
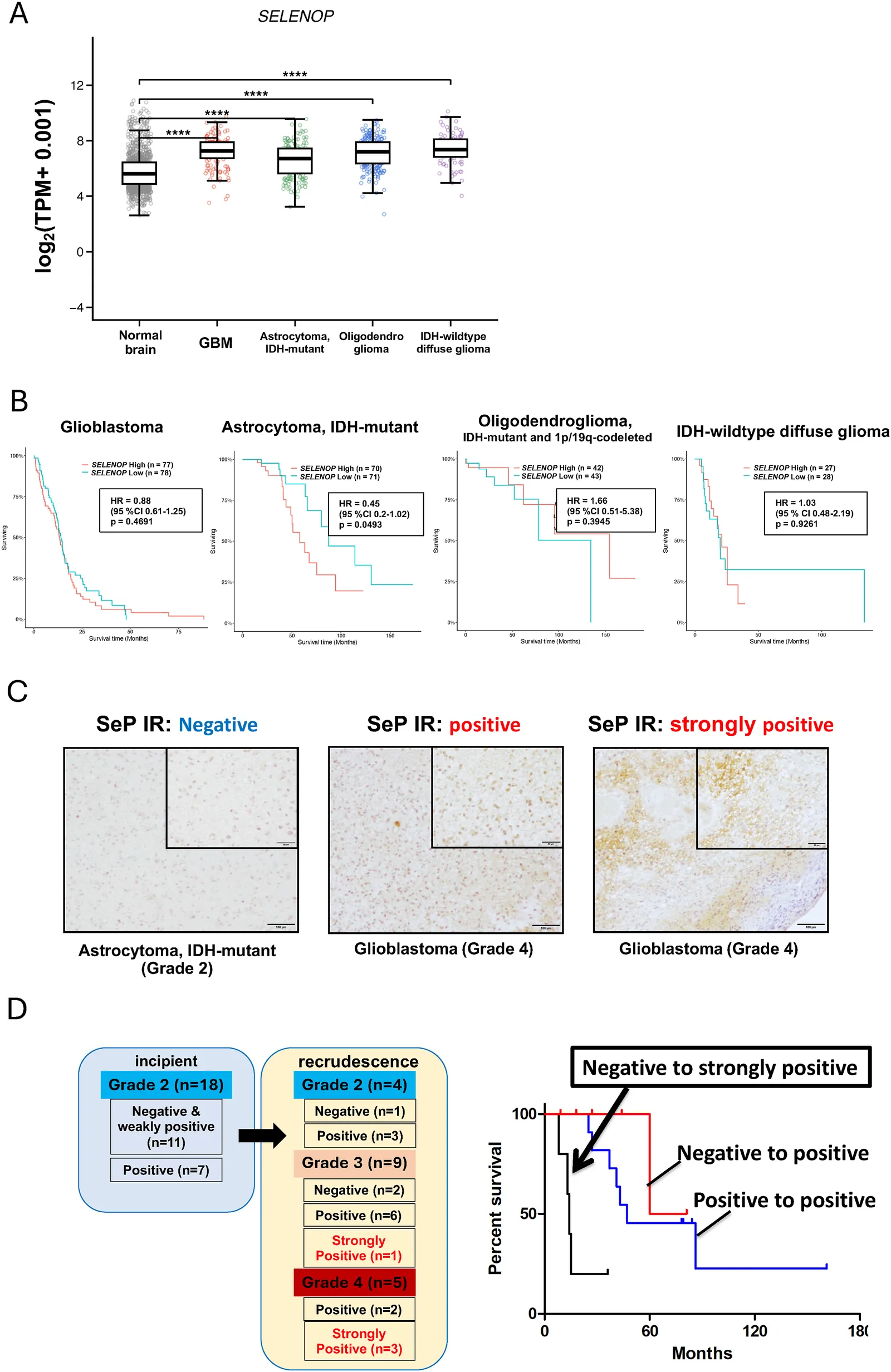
**Selenoprotein P expression is associated with malignant progression and poor prognosis in astrocytoma, IDH-mutant.** **A**, *SELENOP* mRNA expression in glioblastoma (GBM), astrocytoma, IDH-mutant, oligodendroglioma, IDH-mutant and 1p/19q-codeleted, and IDH-wildtype diffuse glioma. RNA-sequencing data from TCGA and GTEx project, uniformly processed using the Toil pipeline, were obtained from the UCSC Xena platform and analyzed using R version 4.5.2. The analyzed cohorts comprised normal brain tissues (n = 1152), glioblastoma (GBM; n = 153), astrocytoma, IDH-mutant (n = 244), oligodendroglioma, IDH-mutant and 1p/19q-codeleted (n = 168), and IDH-wildtype diffuse glioma (n = 94). Gliomas were stratified according to IDH mutation and 1p/19q-codeletion status. **B**, Kaplan–Meier overall survival analysis according to *SELENOP* expression in patients with GBM, astrocytoma, IDH-mutant, oligodendroglioma, IDH-mutant and 1p/19q-codeleted, and IDH-wildtype diffuse glioma. Numbers of patients in each subgroup are shown in the corresponding panels. **C**, Representative immunohistochemical staining of SELENOP in human glioma specimens. Scale bars, 100 μm; inset, 50 μm. **D**, Changes in SELENOP immunoreactivity during malignant progression in paired primary and recurrent astrocytoma, IDH-mutant specimens and the association between recurrent SELENOP immunoreactivity and patient survival. Data are presented as box-and-whisker plots showing the median and interquartile range. Statistical significance was assessed using two-sided Welch's *t*-tests with Holm correction for multiple comparisons (A) or the log-rank test (B and D). ****P < 0.0001.

To further evaluate SELENOP protein expression in clinical glioma specimens, we established an immunohistochemical staining protocol using an anti-human SELENOP antibody (Fig. 5C). We then analyzed paired primary and recurrent tumors obtained from 18 patients initially diagnosed with astrocytoma, IDH-mutant, WHO grade 2. SELENOP immunoreactivity tended to increase with malignant progression, with strongly positive staining observed more frequently in recurrent high-grade astrocytomas (Fig. 5D). Moreover, patients whose recurrent tumors exhibited stronger SELENOP immunoreactivity had significantly poorer clinical outcomes (Fig. 5D). In contrast, SELENOP expression alone was not significantly associated with prognosis in patients with GBM (Supplementary Fig. S11). Together, these findings demonstrate that increased SELENOP expression accompanies malignant progression and is associated with poor clinical outcome in astrocytoma.

To further evaluate the clinical relevance of *CCDC152*, we examined its expression in molecularly classified adult-type diffuse gliomas using RNA-sequencing data from TCGA and GTEx. *CCDC152* expression was significantly higher in GBM and IDH-wildtype diffuse glioma than in normal brain tissues, whereas it was significantly lower in astrocytoma and oligodendroglioma (Fig. 6A). Despite its relatively low overall expression in astrocytoma, elevated *CCDC152* expression within this subgroup was significantly associated with shorter overall survival. By contrast, no significant association was observed in GBM, oligodendroglioma, or IDH-wildtype diffuse glioma (Fig. 6B, Supplementary Fig. S12). These findings indicate that although *CCDC152* expression differs substantially among molecular glioma subtypes, increased *CCDC152* expression identifies a subset of astrocytoma with poor clinical outcome.

**Fig. 6:**
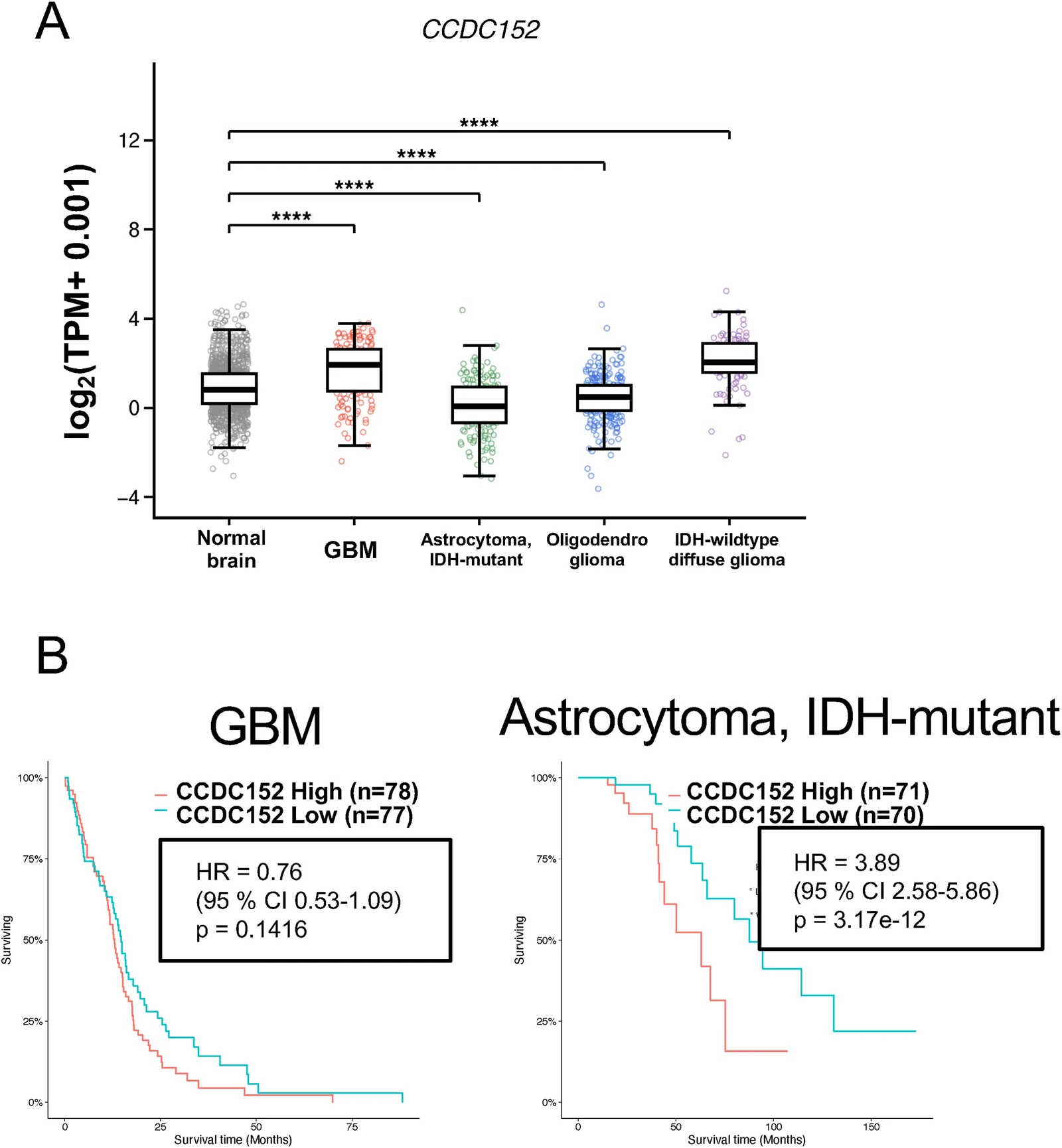
**Elevated *CCDC152* expression is associated with poor prognosis in astrocytoma, IDH-mutant.** **A,***CCDC152* expression in glioblastoma (GBM), astrocytoma, IDH-mutant, oligodendroglioma, IDH-mutant and 1p/19q-codeleted, and IDH-wildtype diffuse glioma. RNA-sequencing data from TCGA and GTEx project, uniformly processed using the Toil pipeline, were obtained from the UCSC Xena platform and analyzed using R version 4.5.2. The analyzed cohorts comprised normal brain tissues (n = 1152), glioblastoma (GBM; n = 153), astrocytoma, IDH-mutant (n = 244), oligodendroglioma, IDH-mutant and 1p/19q-codeleted (n = 168), and IDH-wildtype diffuse glioma (n = 94). Gliomas were stratified according to IDH mutation and 1p/19q-codeletion status. **B,** Kaplan–Meier overall survival analysis according to *CCDC152* expression in patients with GBM and astrocytoma, IDH-mutant. Numbers of patients in each subgroup are shown in the corresponding panels. Statistical significance was assessed using two-sided Welch's *t*-tests with Holm correction for multiple comparisons (A) or the log-rank test (B). ****P < 0.0001.

Together, these findings identify *CCDC152* as a key upstream regulator of the tumor-associated SELENOP cycle, linking SELENOP production, LRP8-mediated selenium uptake, and selenoprotein homeostasis to GBM proliferation and ferroptosis resistance (Fig. 7). Clinical analyses further suggest that elevated *CCDC152* and SELENOP expression is associated with aggressive disease behavior and poor prognosis in astrocytoma, IDH-mutant.

**Fig. 7:**
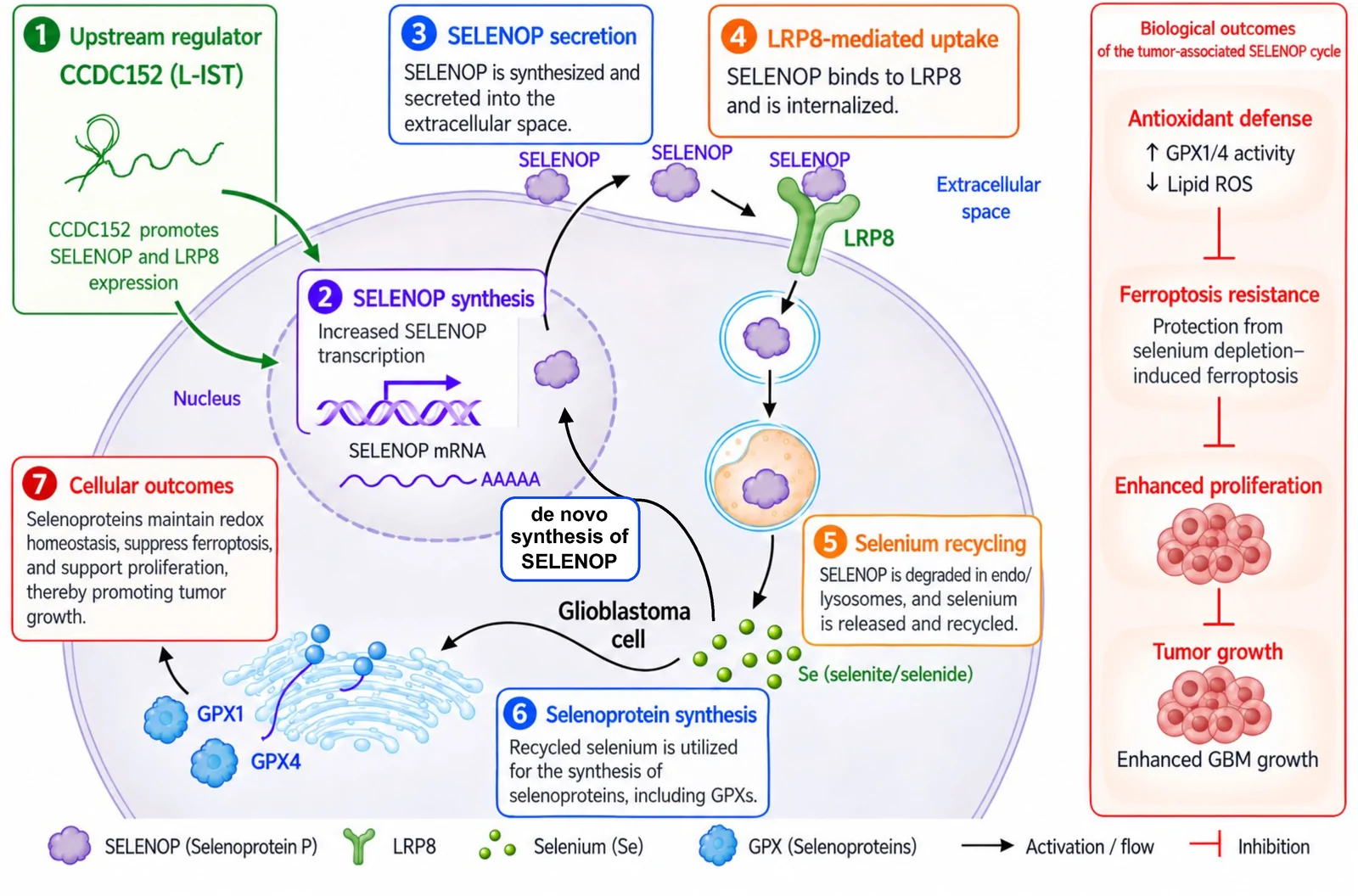
**Proposed model of the tumor-associated SELENOP cycle coordinated by lncRNA *CCDC152*.** lncRNA *CCDC152* promotes SELENOP and LRP8 expression, thereby establishing a tumor-associated SELENOP cycle in GBM. Tumor-derived SELENOP is secreted, recaptured through LRP8, and degraded to release selenium for intracellular selenoprotein synthesis. Recycled selenium also supports de novo SELENOP biosynthesis and secretion, thereby closing the cycle and dynamically sustaining selenium-dependent redox homeostasis. This cycle promotes GBM proliferation, ferroptosis resistance, and tumor growth.

## 4 Discussion

The present study identifies a previously unrecognized selenium utilization system operating in GBM. We demonstrate that highly proliferative GBM cells establish a tumor-associated SELENOP cycle coordinated by *CCDC152*, in which tumor-derived SELENOP is recaptured through LRP8 to sustain intracellular selenoprotein abundance and proliferation. Our findings further reveal an essential role for the intracellular domain of LRP8 and establish the CCDC152–SELENOP–LRP8 axis as an integrated selenium utilization system in GBM (Fig. 7). Consistent with these mechanistic findings, clinical analyses showed that elevated *SELENOP* and *CCDC152* expression was associated with poor prognosis in astrocytoma, IDH-mutant.

Physiologically, the SELENOP cycle enables tissues, particularly the central nervous system, to retain and redistribute selenium independently of liver-derived SELENOP [14], [15], [16]. Our findings suggest that GBM cells exploit this physiological mechanism to establish a tumor-associated selenium utilization system that maintains intracellular selenium availability through SELENOP production, LRP8-mediated uptake, and reutilization. This model provides a plausible explanation for our previous observation that SELENOP depletion reduces GPX1 and GPX4 expression and enhances ferroptosis sensitivity [15]. Clinically, elevated *SELENOP* expression was associated with poor prognosis in astrocytoma, IDH-mutant, suggesting that selenium-related metabolic pathways may also be relevant to aggressive disease behavior in this glioma subtype. This interpretation is further supported by previous studies linking elevated SELENOS and SLC7A11 expression to poor prognosis and ferroptosis resistance in glioma [5], [28], [29]. Together, these findings support a model in which the tumor-associated SELENOP cycle sustains selenium-dependent redox homeostasis and promotes glioma progression under oxidative stress.

Notably, the SELENOP species detected in the conditioned medium of T98G cells migrated at a lower apparent molecular mass than full-length SELENOP. Because the C-terminal region of SELENOP is required for LRP8 binding, whereas N-terminal fragments show little or no receptor-binding activity [30], [31], the detected SELENOP species may represent an N-terminal fragment that is inefficiently recognized by LRP8. Conversely, receptor-competent SELENOP species retaining the C-terminal region may be rapidly internalized and therefore remain scarcely detectable in the extracellular medium. We also detected a high-molecular-mass form of LRP8, which may correspond to a glycosylated receptor species associated with efficient SELENOP uptake [13]. Together, these observations are consistent with preferential capture of receptor-competent SELENOP by GBM cells, thereby reinforcing the tumor-associated SELENOP cycle.

In addition to mediating selenium uptake, LRP8 functions as a ligand-responsive signaling receptor whose abundance and growth-promoting activity are regulated by extracellular SELENOP [19], [32]. Consistent with ligand-dependent receptor trafficking [13], [21], moderate SELENOP exposure promoted GBM cell proliferation, whereas higher SELENOP concentrations reduced LRP8 abundance and attenuated proliferation, suggesting that excessive ligand exposure enhances receptor internalization and degradation. The growth-inhibitory effect of a SELENOP-specific antibody further supports the requirement for productive SELENOP–LRP8 interaction in rapidly proliferating GBM cells. Together, these findings indicate that GBM proliferation depends on appropriate ligand–receptor engagement rather than simply the abundance of SELENOP or LRP8. Although the intracellular domain of LRP8 is essential for its growth-promoting activity, the downstream signaling mechanism remains unresolved. Future studies will be required to determine how ligand binding activates intracellular signaling through the CCDC152–SELENOP–LRP8 axis.

Perhaps the most unexpected finding of the present study is that *CCDC152* promotes, rather than suppresses, the tumor-associated SELENOP cycle in GBM. We previously showed that *CCDC152* acts as an antisense lncRNA that directly interacts with *SELENOP* mRNA and inhibits its translation by interfering with SBP2-mediated SECIS recognition in hepatocyte-derived cells [22]. In contrast, the present study demonstrates that *CCDC152* positively regulates SELENOP production, LRP8 expression, intracellular selenoprotein abundance, and tumor growth in GBM. Consistent with these functional effects, elevated *CCDC152* expression was associated with poor outcome in astrocytoma, IDH-mutant, supporting the clinical relevance of this regulatory pathway. These apparently opposing functions are consistent with the context-dependent nature of lncRNAs, whose activities are shaped by the surrounding molecular environment [33]. In GBM, *CCDC152* may regulate *SELENOP* expression through context-dependent mechanisms acting at the mRNA and translational levels, while its effect on LRP8 expression remains mechanistically unclear. Consequently, *CCDC152* may coordinate both SELENOP production and receptor-mediated selenium uptake. Although EGCg has been identified as an inducer of *CCDC152/L-IST* [22], the endogenous promoter, transcript processing, and upstream regulatory mechanisms controlling *CCDC152* expression remain incompletely understood. Future studies examining the subcellular localization of *CCDC152*, its interacting RNA- and protein-binding partners, and its regulation of LRP8 expression will be required to define these mechanisms. Together with the limited selenium-related phenotype of *Ccdc152*-deficient mice [23], our findings indicate that *CCDC152* is not a universal inhibitor of SELENOP translation but a cell type-dependent regulator whose function is redirected toward coordinated activation of the SELENOP cycle in GBM.

Our findings identify the tumor-associated SELENOP cycle as a potential therapeutic vulnerability for sensitizing GBM cells to ferroptosis. Unlike current strategies that directly inhibit GPX4 or deplete glutathione [4], [18], disruption of the SELENOP cycle would target selenium utilization upstream of selenoprotein biosynthesis, thereby compromising multiple selenium-dependent antioxidant systems [34]. In principle, inhibition of SELENOP production, LRP8-mediated selenium uptake, or *CCDC152* could reduce intracellular selenium availability, impair selenoprotein synthesis, and enhance ferroptosis susceptibility. However, because the SELENOP–LRP8 pathway is essential for physiological selenium delivery to the central nervous system, systemic inhibition of SELENOP or LRP8 could compromise normal brain selenium homeostasis. In contrast, *CCDC152* may represent a more selective therapeutic target. Although *CCDC152* promotes SELENOP production, LRP8 expression, selenoprotein abundance, and tumor growth in GBM, *Ccdc152* deficiency produces only limited selenium-related phenotypes in mice [23], suggesting a relatively minor role in basal selenium homeostasis. Together with its association with poor clinical outcome in astrocytoma, IDH-mutant, these findings support *CCDC152* as a promising target for disrupting the tumor-associated SELENOP cycle. Although efficient delivery to infiltrative GBM remains a challenge, the limited physiological phenotype of *Ccdc152* deficiency provides a rationale for developing *CCDC152*-directed therapeutic strategies.

An intriguing clinical observation of the present study is that a significant association between *SELENOP* mRNA expression and overall survival was detected only in astrocytoma, IDH-mutant, within the transcriptomic datasets analyzed. However, this finding does not exclude the clinical relevance of SELENOP in other glioma subtypes, because SELENOP protein abundance may not directly parallel mRNA expression owing to regulation by selenium availability and the selenoprotein biosynthetic machinery. Moreover, because SELENOP is a secreted protein, its measurement in serum or cerebrospinal fluid may provide complementary biomarker information that is not captured by tumor transcriptomic analysis. Such measurements were not available in the present study and should be addressed in future studies. The association of higher *SELENOP* expression with poor outcome, together with increased SELENOP immunoreactivity in recurrent astrocytoma, suggests that selenium-related metabolic adaptation may accompany aggressive disease behavior in this subtype. However, the present study does not establish functional activation of the tumor-associated SELENOP cycle in astrocytoma, IDH-mutant. Disease progression remains a major clinical challenge in astrocytoma, IDH-mutant, despite recent advances in IDH-targeted therapies, and the molecular mechanisms underlying progression and therapeutic vulnerability remain incompletely defined [35], [36], [37]. Our findings suggest that selenium-related metabolic adaptation may accompany aggressive disease behavior in this subtype. However, whether a functional CCDC152–SELENOP–LRP8 cycle operates in astrocytoma, IDH-mutant, remains to be determined.

Several important questions remain to be addressed. First, although our data support secretion, LRP8-mediated recapture, and intracellular reutilization of tumor-derived SELENOP, direct tracing of selenium flux through the SELENOP cycle will be required to define its quantitative contribution to GBM selenium metabolism. Second, although the intracellular domain of LRP8 is essential for its growth-promoting activity, the downstream signaling mechanism remains unclear, including the relative contribution of Wnt/β-catenin signaling and other adaptor-dependent pathways. Third, the molecular mechanism by which *CCDC152* coordinately regulates SELENOP and LRP8 expression remains to be elucidated. In addition, the identities of the lower-molecular-mass SELENOP species and the slowly migrating LRP8 form require further biochemical characterization. Another limitation is that the selenium content and Sec occupancy of SELENOP purified from pooled donor plasma were not directly determined. Finally, it will be important to determine whether the tumor-associated SELENOP cycle represents a glioma-specific adaptation or a more general mechanism by which malignant cells optimize selenium utilization under oxidative stress. Addressing these questions will further clarify the role of selenium metabolism in tumor progression and facilitate the development of therapeutic strategies targeting the CCDC152–SELENOP–LRP8 axis.

In conclusion, our study identifies a previously unrecognized tumor-associated SELENOP cycle that enables GBM cells to coordinate selenium acquisition, intracellular reutilization, and selenoprotein maintenance. We further identify *CCDC152* as an upstream coordinator of this pathway and demonstrate that the intracellular domain of LRP8 is essential for its growth-promoting activity. Together, these findings establish the CCDC152–SELENOP–LRP8 axis as an integrated selenium utilization system linking selenium metabolism to GBM progression and highlight it as a potential therapeutic target.

## CRediT authorship contribution statement

**Stephanie Siu:** Formal analysis, Data curation. **Hiroki Taguchi:** Formal analysis, Data curation. **Xi Zheng:** Formal analysis, Data curation. **Hikari Sugiura:** Formal analysis, Data curation. **Shota Yamashita:** Writing – review & editing, Formal analysis, Data curation. **Tatsuya Takahashi:** Formal analysis, Data curation. **Masato Kosuge:** Formal analysis, Data curation. **Takayuki Kaneko:** Formal analysis, Data curation. **Misaki Shimizu:** Formal analysis, Data curation. **Yoshiteru Shimoda:** Resources, Formal analysis, Data curation. **Kota Noritsugu:** Formal analysis. **Kotoko Arisawa:** Formal analysis, Data curation. **Mika Watanabe:** Formal analysis, Data curation. **Takashi Toyama:** Writing – review & editing, Funding acquisition, Conceptualization. **Masayuki Kanamori:** Supervision, Resources, Investigation, Conceptualization. **Hidenori Endo:** Supervision, Resources, Conceptualization. **Yoshiro Saito:** Writing – review & editing, Writing – original draft, Supervision, Project administration, Funding acquisition, Formal analysis.

## Ethics declaration

Written informed consent to take part in the study and to publish the article has been obtained from all participants or their legal representatives. The privacy rights of participants have been observed.

This study included organ or tissue donors. This study includes human biological material and consent was obtained by donors, or their next of kin or legal representatives, for use in this study and for publication of the article. The samples used in this research were not sourced from executed prisoners or prisoners of conscience.

This study was performed in compliance with relevant laws, regulatory frameworks and guidelines where the research took place. This study was approved by the Institutional Review Board of Tohoku University. (Approval No. 2026-1-309)

This study was conducted in accordance with the ARRIVE (Animal Research: Reporting of In Vivo Experiments) guidelines. This study was approved by the Animal Care and Use Committee of Tohoku University. (Approval No. 2022-005-03)

## Declaration of generative AI and AI-assisted technologies in the manuscript preparation process

During the preparation of this work, the authors used ChatGPT (OpenAI) to assist with language editing and manuscript organization. After using this tool, the authors reviewed and edited the content as needed and take full responsibility for the content of the publication.

## Declaration of competing interest

The authors declare that they have no known competing financial interests or personal relationships that could have appeared to influence the work reported in this paper.

## Data Availability

Data will be made available on request.
