# Supplementary material for: LncRNA CCDC152 coordinates a tumor-associated selenoprotein P cycle that sustains selenium-dependent redox homeostasis in glioblastoma
Source: Redox Biol. 2026 Oct 12;98:104438. doi: 10.1016/j.redox.2026.104438 (PMC13683443; doi:10.1016/j.redox.2026.104438)
Supplement: Supplementary file 1 [file mmc1.docx]

**Supplemental Table S1. Clinicopathological characteristics of patient-derived glioblastoma cell lines (PD-GBM) used in this study.**

| Cell line | PD-GBM1 | PD-GBM2 | PD-GBM3 |
| --- | --- | --- | --- |
| Age | 69 | 43 | 66 |
| Sex | M | M | M |
| Diagnosis | Glioblastoma, IDH-wildtype, CNS WHO grade 4 | Glioblastoma, IDH-wildtype, CNS WHO grade 4 | Glioblastoma, IDH-wildtype, CNS WHO grade 4 |
| MGMT | Unmethylated | Methylated | Methylated |
| MIB-1 index | 34% | 31.8% | 24.4% |
| EOR | GTR | GTR | GTR |
| OS | 9 months  (dead) | 42 months (alive) | 17 months  (dead) |

Three primary GBM cell lines (PD-GBM1, PD-GBM2, and PD-GBM3) were established from surgically resected glioblastoma, IDH-wildtype (CNS WHO grade 4), specimens obtained at Tohoku University Hospital. Clinicopathological information includes patient age, sex, pathological diagnosis, the promoter of O6-methylguanine–methyltransferase gene (MGMTp) methylation status, Ki-67 (MIB-1) labeling index, extent of resection (EOR), and overall survival (OS).

**Supplemental Table S2. Primers used in the present study.**

| Primer |  | Sequence |
| --- | --- | --- |
| Human GAPDH | F | GCACCGTCAAGGCTGAGAAC |
|  | R | TGGTGAAGACGCCAGTGGA |
| Human SELENOP | F | CCCCCAGCCTGGAGCATAAG |
|  | R | TGCACAGGTATCAGCTGGCTT |
| Human GPX1 | F | CAGTCGGTGTATGCCTTCTCG |
|  | R | GAGGGACGCCACATTCTCG |
| Human GPX4 | F | GAGGCAAGACCGAAGTAAACTAC |
|  | R | CCGAACTGGTTACACGGGAA |
| Human LRP8 | F | GTTGCCACCAATCGCATCT |
|  | R | TCGGGTCACTGGCCTTGT |
| Human CCDC152 | F | GGGGAACTAGGAGCAACAGC |
|  | R | AGACCTCCTTTGCTTGCATT |

**Supplemental Table S3. Antibodies used in the present study.**

| <Primary antibodies> | Catalog No |
| --- | --- |
| Rat monoclonal SELENOP (human specific) BD1 antibody | Original [25] |
| Rat monoclonal SELENOP (human specific) BD3 antibody | Original [25] |
| Rat monoclonal SELENOP (human specific) AA3 antibody | Original [25] |
| Rat monoclonal SELENOP (human specific) AH5 antibody | Original [25] |
| Mouse monoclonal Anti-GAPDH Peroxidase conjugated | 015-25473, Wako |
| Rabbit monoclonal Anti-ApoER2 | ab108208, abcam |
| Rabbit monoclonal Anti-GPX1 antibody | ab22604, abcam |
| Rabbit monoclonal Anti-GPX4 antibody | ab125066, abcam |
| Mouse monoclonal Anti-β-actin | clone AC-15, Sigma |
| Rabbit monoclonal Anti-TRXR1 antibody | #15140, Cell Signaling Technology |
| Mouse monoclonal Anti-human Ki-67 antibody | clone MIB-1, Agilent Technologies |
|  |  |
| <Secondary antibodies> | Catalog No |
| Goat polyclonal Anti-Rabbit Immunoglobulins/HRP | 20073563, Dako |
| Goat polyclonal Anti-Rat Immunoglobulins/HRP | XGO1523121, R&D Systems |
| Goat polyclonal Anti-Mouse Immunoglobulins/HRP | 20051789 Dako |
| Streptavidin/HRP | 41386930, Dako |

**Supplemental Table S4. siRNAs used in the present study.**

| siRNA |  | Target sequence |
| --- | --- | --- |
| si SELENOP #1 | F | GAGAUAUGCCAGCAAGUGA |
|  | R | UCACUUGCUGGCAUAUCUC |
| si SELENOP #2 | F | GCAUAUUCCUGUUUAUCAA |
|  | R | UUGAUAAACAGGAAUAUGC |
| si SELENOP #3 | F | GCAUACUGCAGGCAUCUAA |
|  | R | UUAGAUGCCUGCAGUAUGC |
| si LRP8 #1 | F | CGCUGAUCUCCUCCACUGA |
|  | R | UCAGUGGAGGAGAUCAGCG |
| si LRP8 #2 | F | GUGACCUCUCCUACCGUAA |
|  | R | UUACGGUAGGAGAGGUCAC |
| si LRP8 #3 | F | GACCUACUGACCAAGAACU |
|  | R | AGUUCUUGGUCAGUAGGUC |
| si CCDC152 #1 | F | ACAAGGAGAUUGCAAUUCUUCGUAA |
|  | R | UUACGAAGAAUUGCAAUCUCCUUGU |
| si CCDC152 #2 | F | GCUACAACAGACCAUUGAA |
|  | R | UUCAAUGGUCUGUUGUAGC |

**Supplementary Figure S1. Expression of members of the LDL receptor family in glioma cell lines.** Relative mRNA expression levels of *LRP1* (left) and *LRP2* (right) in human glioma cell lines and reference cancer cell lines were determined by quantitative real-time PCR. Expression levels were normalized to *GAPDH*. Data are presented as mean ± SD from three independent experiments.

**Supplementary Figure S2. SELENOP mRNA expression in patient-derived GBM cell lines (PD-GBM) (Left) and Cell proliferation of PD-GBM for 72 h (Right).** Data are presented as mean ± SD from at least three independent experiments. Statistical significance was determined using one-way ANOVA followed by Tukey's multiple-comparison test. **P < 0.01 and ***P < 0.001.

**Supplementary Figure S3. Effects of selenium supplementation and SELENOP overexpression on GBM cell proliferation. A**, Relative *SELENOP* mRNA expression in YKG1 and SELENOP-overexpressing YKG1 cells cultured for 24 h, as determined by quantitative real-time PCR. Expression levels were normalized to *GAPDH*. **B,** Immunoblot analysis of SELENOP secreted into conditioned medium from YKG1 and SELENOP-overexpressing YKG1 cells cultured in the absence or presence of 100 nM sodium selenite. Coomassie Brilliant Blue (CBB) staining is shown as a loading control. **C,** Cell proliferation of YKG1 and SELENOP-overexpressing YKG1 cells cultured in the absence or presence of 100 nM sodium selenite for the indicated period. Data are presented as mean ± SD from three independent experiments. Statistical significance was determined using two-way ANOVA followed by Tukey’s multiple-comparison test (**C**). ****P < 0.0001.

**Supplementary Figure S4. LRP8 depletion reduces GPX1 and GPX4 mRNA expression in GBM cells.** Relative mRNA expression levels of GPX1 and GPX4 in T98G cells transfected with a non-targeting control siRNA or three independent siRNAs targeting *LRP8* were determined by quantitative real-time PCR. Expression levels were normalized to *GAPDH*. Data are presented as mean ± SD from three independent experiments. Statistical significance was determined using one-way ANOVA followed by Tukey’s multiple-comparison test. *P < 0.05.

**Supplementary Figure S5. Independent siRNAs targeting LRP8 consistently suppress GBM cell proliferation.** Cell proliferation of T98G cells transfected with a non-targeting control siRNA or three independent siRNAs targeting *LRP8* was evaluated at 24, 48, and 72 h after transfection. Cell growth is expressed relative to that of siControl-transfected cells at 24 h, which was set to 100%. Data are presented as mean ± SD from three independent experiments. Statistical significance was determined using two-way ANOVA followed by Tukey’s multiple-comparison test. *P < 0.05, **P < 0.01, ***P < 0.001, and ****P < 0.0001.

**Supplementary Figure S6. Knockdown of LRP8 or CCDC152 suppresses subcutaneous tumor growth in vivo. A,** Representative photograph of subcutaneous xenograft tumors established from control (siControl) and *LRP8*-knockdown T98G cells at the experimental endpoint. **B,** Tumor growth of subcutaneous xenografts established from control and *LRP8*-knockdown T98G cells. Tumor size was measured at the indicated time points after xenograft implantation. **C,** Growth of subcutaneous xenografts established from control and *CCDC152*-knockdown T98G cells. Tumor size was measured at the indicated time points after xenograft implantation. Data are presented as individual animals. Statistical significance at each time point was evaluated using one-way ANOVA followed by Tukey's multiple-comparison test. * P < 0.05, ** P < 0.01.

**Supplementary Figure S7. Enlarged UMAP visualization of CCDC152 expression in human gliomas.**

Enlarged view corresponding to Fig. 4A. Upper panel, UMAP visualization colored according to the primary diagnosis labels as originally annotated in the source dataset. Lower panel, relative *CCDC152* expression levels visualized on the same UMAP, with red and blue indicating high and low expression, respectively.

**Supplementary Figure S8. *CCDC152* functions as a non-coding RNA in GBM cells. A**, Expression of *CCDC152 and iNOS-HA* mRNAs in T98G cells 24 h after transfection with empty vector (Plasmid-Con), HA-tagged *iNOS* expression vector (positive control), or HA-tagged *CCDC152* expression vector. Transcript levels were determined by quantitative real-time PCR. **B**, Immunoblot analysis using an anti-HA antibody. A distinct HA-tagged iNOS protein band was readily detected, whereas no detectable HA-tagged CCDC152 protein was observed despite robust *CCDC152* expression. CBB staining is shown as a loading control. Data are presented as mean ± SD from three independent experiments.

**Supplementary Figure S9. Differential expression of *LRP8* in molecularly classified diffuse gliomas.** *LRP8* mRNA expression in glioblastoma (GBM), astrocytoma, IDH-mutant, oligodendroglioma, IDH-mutant and 1p/19q-codeleted, and IDH-wildtype diffuse glioma compared with normal brain tissues. RNA-sequencing data from TCGA and GTEx, uniformly processed using the Toil pipeline, were obtained through the UCSC Xena platform and analyzed using R. Tumors were classified according to the WHO CNS5 molecular classification. Box-and-whisker plots show the median, interquartile range, and minimum and maximum values. Each dot represents an individual patient sample. Statistical significance was assessed using two-sided Welch's *t*-tests with Holm correction for multiple comparisons. ****P < 0.0001.

**Supplementary Figure S10. Prognostic significance of *LRP8* expression in molecularly classified diffuse gliomas.** Kaplan–Meier overall survival analyses according to *LRP8* expression in patients with glioblastoma (GBM), astrocytoma, IDH-mutant, oligodendroglioma, IDH-mutant and 1p/19q-codeleted, and IDH-wildtype diffuse glioma. Patients were stratified into high- and low-expression groups using the median expression level as the cutoff. Statistical significance was evaluated using the log-rank test.

**Supplementary Figure S11. Association between SELENOP immunoreactivity and patient survival.** Kaplan–Meier overall survival analysis of patients with glioblastoma according to SELENOP immunohistochemical staining intensity. Tumors were classified as negative, positive, or strongly positive based on SELENOP immunoreactivity. Statistical significance was evaluated using the log-rank test.

**Supplementary Figure S12. Kaplan–Meier overall survival analysis according to *CCDC152* expression.** Kaplan–Meier overall survival analyses according to *CCDC152* expression in patients with oligodendroglioma, IDH-mutant and 1p/19q-codeleted (left) and IDH-wildtype diffuse glioma (right). Statistical significance was evaluated using the log-rank test.
